# Long Non-Coding RNAs *SNHG7*, *SNHG12*, and *SNHG14* in Rheumatoid Arthritis: Clinical and In Silico Analysis

**DOI:** 10.3390/cimb48090860

**Published:** 2026-08-25

**Authors:** Michał Czerewaty, Paweł Dec, Krzysztof Safranow, Andrzej Pawlik

**Affiliations:** 1 Department of Physiology, Pomeranian Medical University, 70-111 Szczecin, Poland; michal.czerewaty@pum.edu.pl (M.C.); pawel_dec@onet.pl (P.D.); 2 Department of Biochemistry and Medical Chemistry, Pomeranian Medical University, 70-111 Szczecin, Poland; chrissaf@mp.pl

**Keywords:** lncRNA, SNHGs, rheumatoid arthritis, synovial membrane, expression

## Abstract

Rheumatoid arthritis (RA) is an autoimmune disease with a complex pathogenesis. It has been shown that epigenetic modifications can influence the differentiation and function of many immune system cells involved in the pathogenesis of RA. Important epigenetic factors include non-coding RNAs, such as long non-coding RNAs (lncRNAs). Non-coding RNAs can influence numerous immune processes in RA. This study aimed to evaluate the expression of lncRNAs *SNHG7*, *SNHG12*, and *SNHG14* in the synovial membranes of patients with RA and to correlate these results with selected clinical parameters of the disease. This study included 46 patients diagnosed with RA and 67 healthy individuals as the control group. The expression levels of the lncRNAs *SNHG7*, *SNHG12*, and *SNHG14* in synovial membranes in patients with RA were statistically higher than those in the control group. The synovial expression of *SNHG14* showed a statistically significant negative correlation with C-reactive protein values. There were no statistically significant correlations between synovial expression of *SNHG7*, *SNHG12*, and clinical parameters. The study results suggest that lncRNAs may potentially play a role in the development of RA.

## 1. Introduction

Rheumatoid arthritis (RA) is an autoimmune disease that causes joint damage and leads to the development of numerous extra-articular complications. Many factors, not yet fully understood, contribute to the pathogenesis of this disease. Among the most important are genetic factors and epigenetic modifications, which contribute to abnormalities in the immune response, leading to the development of chronic inflammation within the joints and their destruction [1]. RA is characterised by an abnormal immune response that activates numerous immune cells to secrete a range of pro-inflammatory mediators—chemokines, cytokines, chemotaxis-enhancing factors, and metalloproteinases—which sustain and exacerbate the inflammatory process [2]. This contributes to the development of inflammation within the synovial membrane of the joints and the formation of joint erosions, leading to joint damage [3,4]. Recent studies have demonstrated the role of epigenetic modifications in the pathogenesis of RA. These changes may lead to dysregulation of the immune response, resulting in increased inflammation [5,6]. Currently, researchers are actively seeking new biomarkers that could help predict the risk of developing this disease, monitor disease activity and treatment response, and forecast the onset of RA complications. It has been shown that epigenetic modifications can influence the differentiation and function of many immune system cells involved in the pathogenesis of RA. Important epigenetic factors include non-coding RNAs, such as microRNAs and long non-coding RNAs (lncRNAs). Non-coding RNAs can influence numerous immune processes in RA by regulating fibroblast-like synoviocyte (FLS) function, activating immune cells, and synthesising various chemokines and cytokines [7,8]. LncRNAs are molecules longer than 200 nucleotides. Their role is to regulate the expression of many genes, thereby influencing the synthesis of various proteins, including inflammatory mediators; regulating the expression of transcription factors; and modulating numerous signalling pathways that may contribute to the development of inflammation in RA. In patients with RA, changes in the expression of many types of lncRNAs have been demonstrated both in the immune system and in the synovial membrane of the joints, which may influence the development of chronic synovial inflammation and the formation of joint erosions [9,10]. It is believed that lncRNAs may influence the function of immune system cells and inflammatory changes in the synovial membrane, which affect the course and activity of the disease process in RA. Current research is exploring the use of lncRNAs as diagnostic markers to aid in the prognosis and assessment of disease activity in RA [8,11]. This study aimed to evaluate the expression of the lncRNAs small nucleolar RNA host genes (SNHGs) *SNHG7*, *SNHG12*, and *SNHG14* in the synovial membranes of patients with RA and to correlate these findings with selected clinical parameters of the disease.

## 2. Materials and Methods

### 2.1. Patients

This study included 46 patients diagnosed with RA according to the American College of Rheumatology/European League Against Rheumatism criteria, all of whom underwent metacarpophalangeal joint synovectomy for clinical reasons. The control group comprised 67 healthy individuals with no history of autoimmune or inflammatory diseases who underwent synovectomy of the metacarpophalangeal joints for the treatment of post-traumatic conditions. Patients with symptoms of inflammation in the palm area were excluded from the study. The RA group had a mean age of 58 years (36 females, 10 males), while the control group had a mean age of 56 years (42 females, 25 males). All patient samples were obtained from the Department of Surgery in Szczecin. The analysis of all three lncRNAs (*SNHG7*, *SNHG12*, and *SNHG14*) was performed in the same study group, consisting of 46 patients with RA and 67 control subjects.

### 2.2. RNA Isolation

Collected tissues were immediately stabilised in RNAlater Solution (Invitrogen, Waltham, MA, USA) and stored at −80 °C until further processing. Total RNA was extracted from homogenised samples using the RNeasy Fibrous Tissue Mini Kit (Qiagen, Hilden, Germany) in accordance with the manufacturer’s instructions. RNA concentration and purity were determined by spectrophotometric analysis using a DS-11 FX spectrophotometer (DeNovix, Wilmington, DE, USA).

### 2.3. Real-Time Quantitative Reverse Transcription Polymerase Chain Reaction (RT-qPCR)

For cDNA synthesis, 0.4 µg of total RNA was reverse-transcribed using the RevertAid First Strand cDNA Synthesis Kit (Thermo Scientific, Waltham, MA, USA) following the manufacturer’s protocol. The expression levels of the selected genes (*SNHG7*, *SNHG12*, and *SNHG14*) and the reference gene were determined by RT-qPCR using an ABI PRISM^®^ 7500 Fast Sequence Detection System (Applied Biosystems, Waltham, MA, USA). *RPLP0* was used as the reference gene to normalise mRNA expression across samples [12,13,14,15,16,17]. It has been previously shown to be stable in human synovial tissue and FLSs under inflammatory and RA conditions (mean *RPLP0* Ct values were 20.44 ± 2.26 in the RA group and 20.60 ± 2.33 in the control group).

Real-time PCR conditions were as follows: 95 °C (15 s), followed by 40 cycles at 95 °C (15 s) and 60 °C (1 min). Melting curve analysis confirmed the presence of a single, specific amplification product. Each sample was analysed in two technical replicates. Expression levels were calculated using the 2^−ΔCt^ method. To improve data visualisation, results are presented after log_10_ transformation of 2^−ΔCt^ values.

The following primers were used for gene expression analysis:

*SNHG7* forward: 5′- GTGACTTCGCCTGTGATGGA -3′;
*SNHG7* reverse: 5′- GGCCTCTATCTGTACCTTTATTCC -3′.
*SNHG12* forward: 5′- TCTGGTGATCGAGGACTTCC -3′;
*SNHG12* reverse: 5′- ACCTCCTCAGTATCACACACT -3′.
*SNHG14* forward: 5′- GTGGATGAGGGTGATGCCTATT -3′;
*SNHG14* reverse: 5′- CCAACACCAGTCAATGCACA -3′.
*RPLP0* forward: 5′- TGGTCATCCAGCAGGTGTTCGA -3′;
*RPLP0* reverse: 5′- ACAGACACTGGCAACATTGCGG -3′.

### 2.4. Bioinformatic Analysis

An in silico approach was used to further evaluate the role of lncSNHGs in RA patients. To compare the results of our study with in silico data, we evaluated three public datasets (GSE77298 [18], GSE55235 [19], and GSE55457 [19]) sourced from the Gene Expression Omnibus (GEO) database using the GEOquery R package. The corresponding expression profiling was performed on the Affymetrix Human Genome U133 Plus 2.0 Array platform. For all three GEO datasets, we used only synovial tissue biopsy samples from RA patients and healthy controls. Within these datasets, three specific lncSNHGs (*SNHG7*, *SNHG12*, and *SNHG14*) were targeted for comparative analysis. Only GSE77298 included probes for *SNHG7*, *SNHG12*, and *SNHG14*, whereas GSE55235 and GSE55457 included probes only for *SNHG14*. In silico analyses were performed using R (version 4.5.3) in the RStudio environment (version 2026.01.2). Raw microarray expression data were retrieved and processed using the GEOquery R package, followed by quantile normalisation using the limma R package. For analysis and visualisation, gene expression levels were log_2_-transformed using log_2_(expression + 1). Log_2_ fold changes (LFCs) were estimated using limma based on a linear model with empirical Bayes moderation of standard errors, and fold changes (FCs) were calculated as 2^LFC^. Statistical significance was assessed using the moderated *t*-test for LFC estimates in limma. To address multiple testing, adjusted *p*-values calculated using the Benjamini–Hochberg false discovery rate (FDR) method were also reported. In each dataset, we analysed all available probe IDs for each target gene. Then, we made a separate plot for each individual probe ID.

### 2.5. Statistical Analysis

Statistical analysis was performed using STATISTICA PL software, version 13.1 (StatSoft, Inc., Tulsa, OK, USA). The Shapiro–Wilk W-test was used to assess whether the data followed a normal distribution. Because the data deviated from normality, non-parametric methods were applied. The Mann–Whitney U-test was used for group comparisons, while Spearman’s rank correlation coefficients were calculated to assess correlations within groups. A *p*-value < 0.05 was considered statistically significant.

## 3. Results

**Figure 1:**
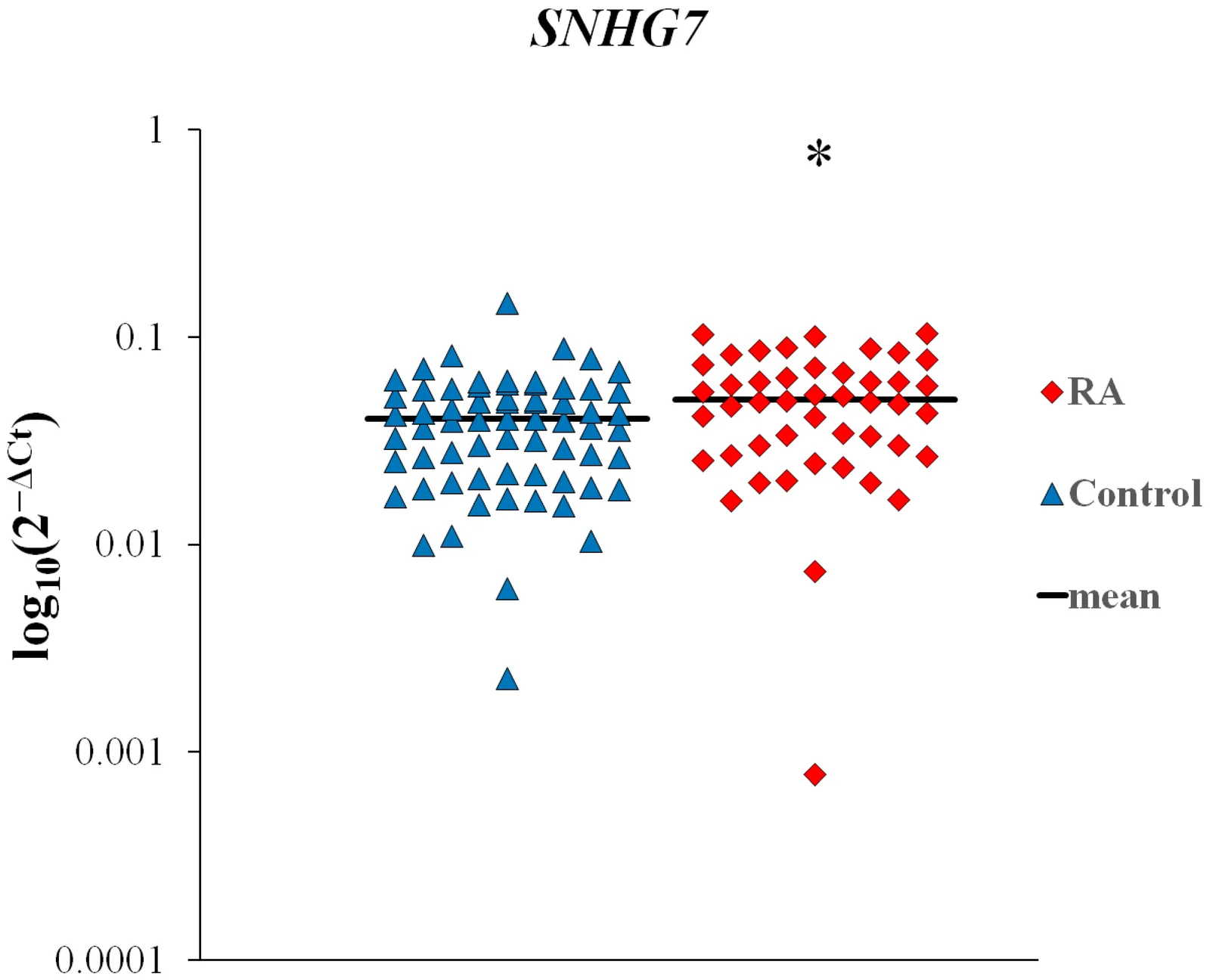
Synovial expression of *SNHG7* in RA patients (*n* = 46) and controls (*n* = 67). Each dot represents an individual sample. * *p* < 0.05, Mann–Whitney U-test.

**Figure 2:**
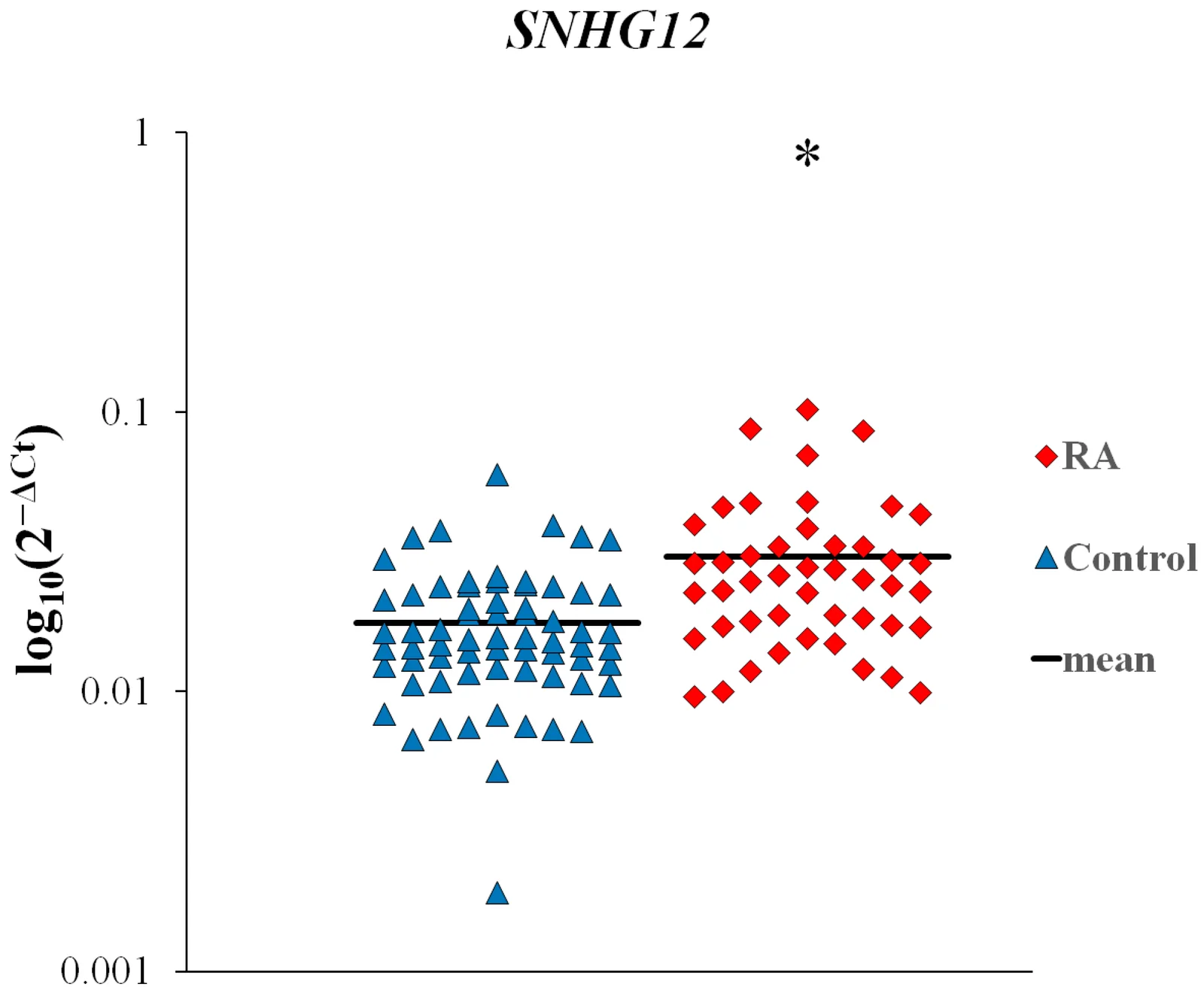
Synovial expression of *SNHG12* in RA patients (*n* = 46) and controls (*n* = 67). Each dot represents an individual sample. * *p* < 0.001, Mann–Whitney U-test.

**Figure 3:**
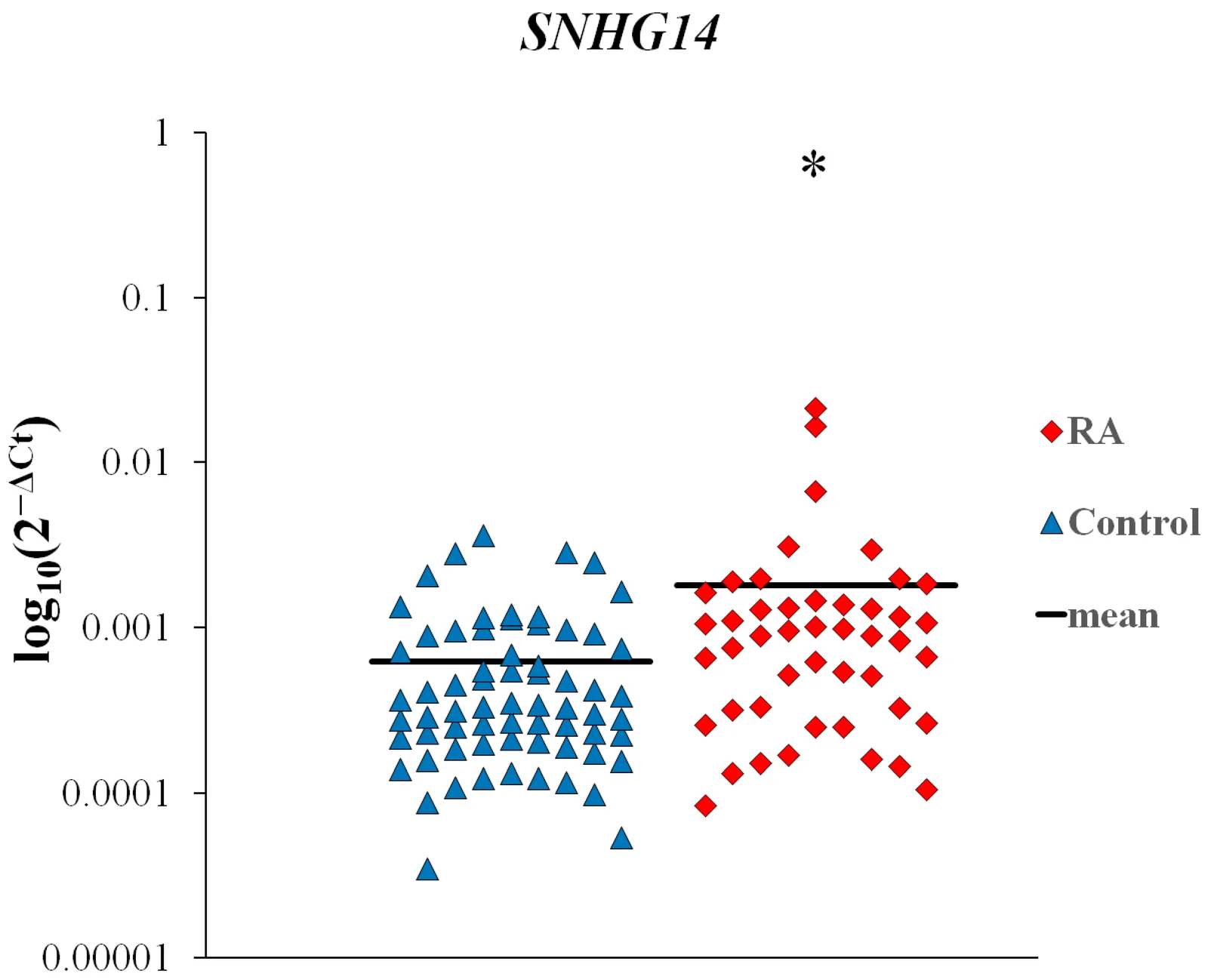
Synovial expression of *SNHG14* in RA patients (*n* = 46) and controls (*n* = 67). Each dot represents an individual sample. * *p* < 0.05, Mann–Whitney U-test.

The expression levels of the lncRNAs *SNHG7*, *SNHG12*, and *SNHG14* in synovial membranes from patients with RA were significantly higher than in the control group (Figure 1, Figure 2 and Figure 3, respectively).

We also compared the synovial expression of *SNHG7*, *SNHG12*, and *SNHG14* between patients who were seropositive and seronegative for rheumatoid factor and anti-cyclic citrullinated peptide antibodies and those with Larsen scores between I–II and III–V. As shown in Supplementary Figures S1–S15, these differences were not statistically significant.

In the next part of the study, we examined the correlations between the synovial expression of *SNHG7*, *SNHG12*, and *SNHG14* and selected clinical parameters. The synovial expression of *SNHG14* showed a statistically significant negative correlation with C-reactive protein (CRP) values (*p* = 0.0063) (Table 1). However, this correlation is not statistically significant after applying the Bonferroni correction (expected *p*-value: 0.0014). There were no statistically significant correlations between synovial expression of *SNHG7* and *SNHG12* and clinical parameters (Table 2 and Table 3, respectively).

### In Silico Analysis

All available probe IDs for *SNHG7*, *SNHG12*, and *SNHG14* were analysed across three datasets: GSE77298, GSE55235, and GSE55457.

**Figure 4:**
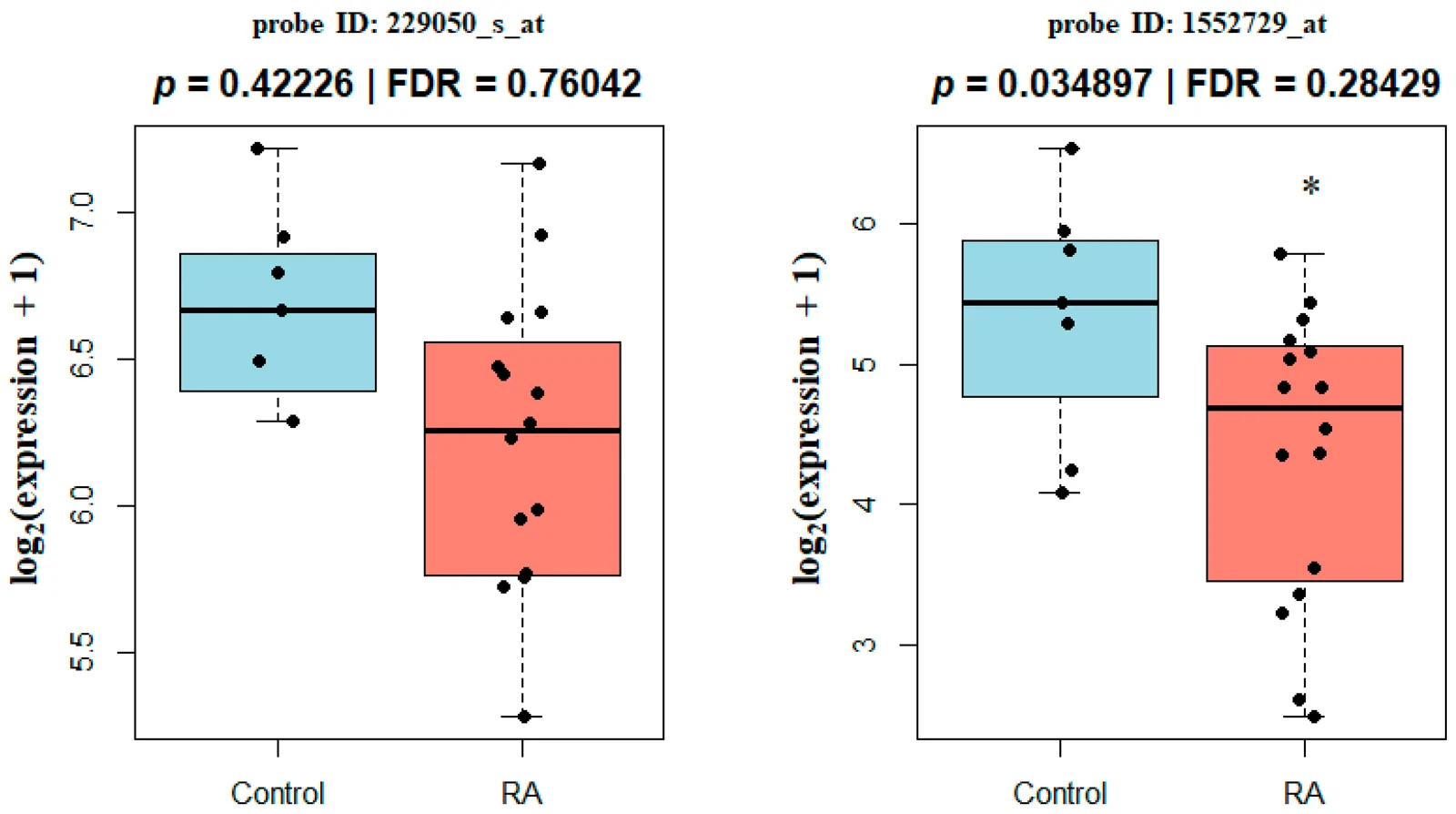
Microarray-based expression profiling of *SNHG7* across all available probe IDs in synovial tissues from RA patients (*n* = 16) and healthy controls (*n* = 7) from the GSE77298 cohort. * *p* < 0.05, empirical Bayes moderated *t*-test.

**Figure 5:**
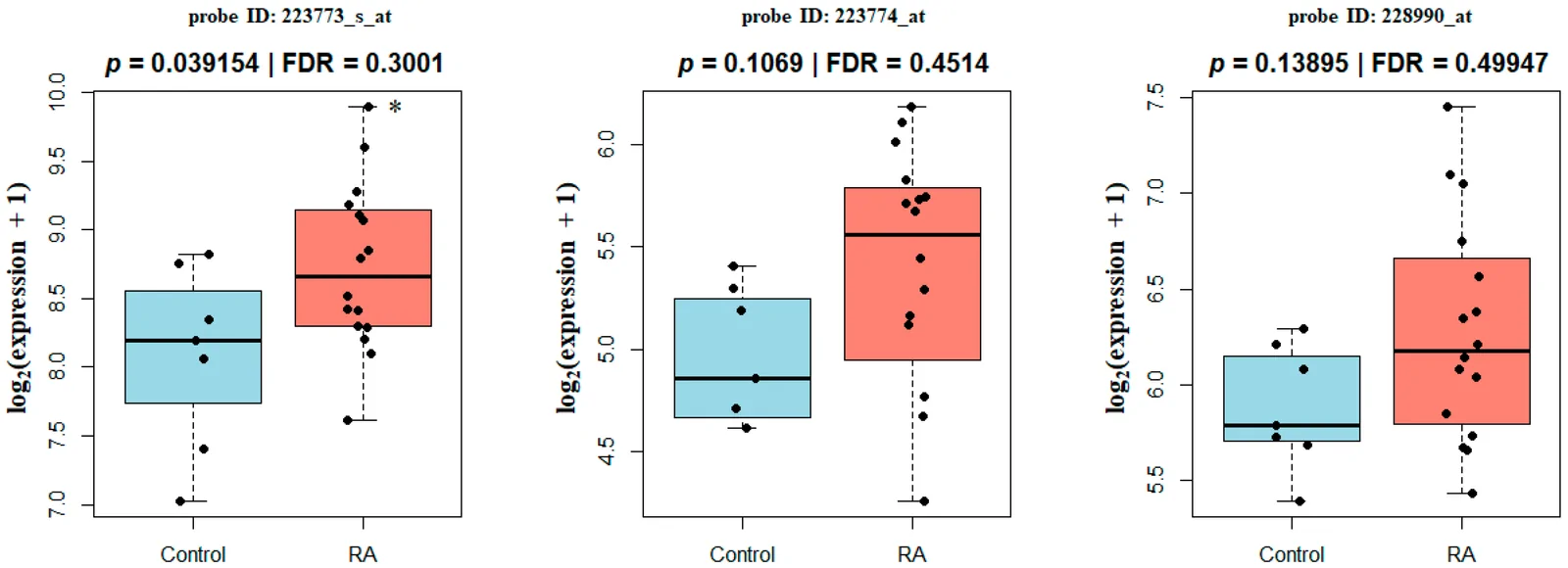
Microarray-based expression profiling of *SNHG12* across all available probe IDs in synovial tissues from RA patients (*n* = 16) and healthy controls (*n* = 7) from the GSE77298 cohort. * *p* < 0.05, empirical Bayes moderated *t*-test.

**Figure 6:**
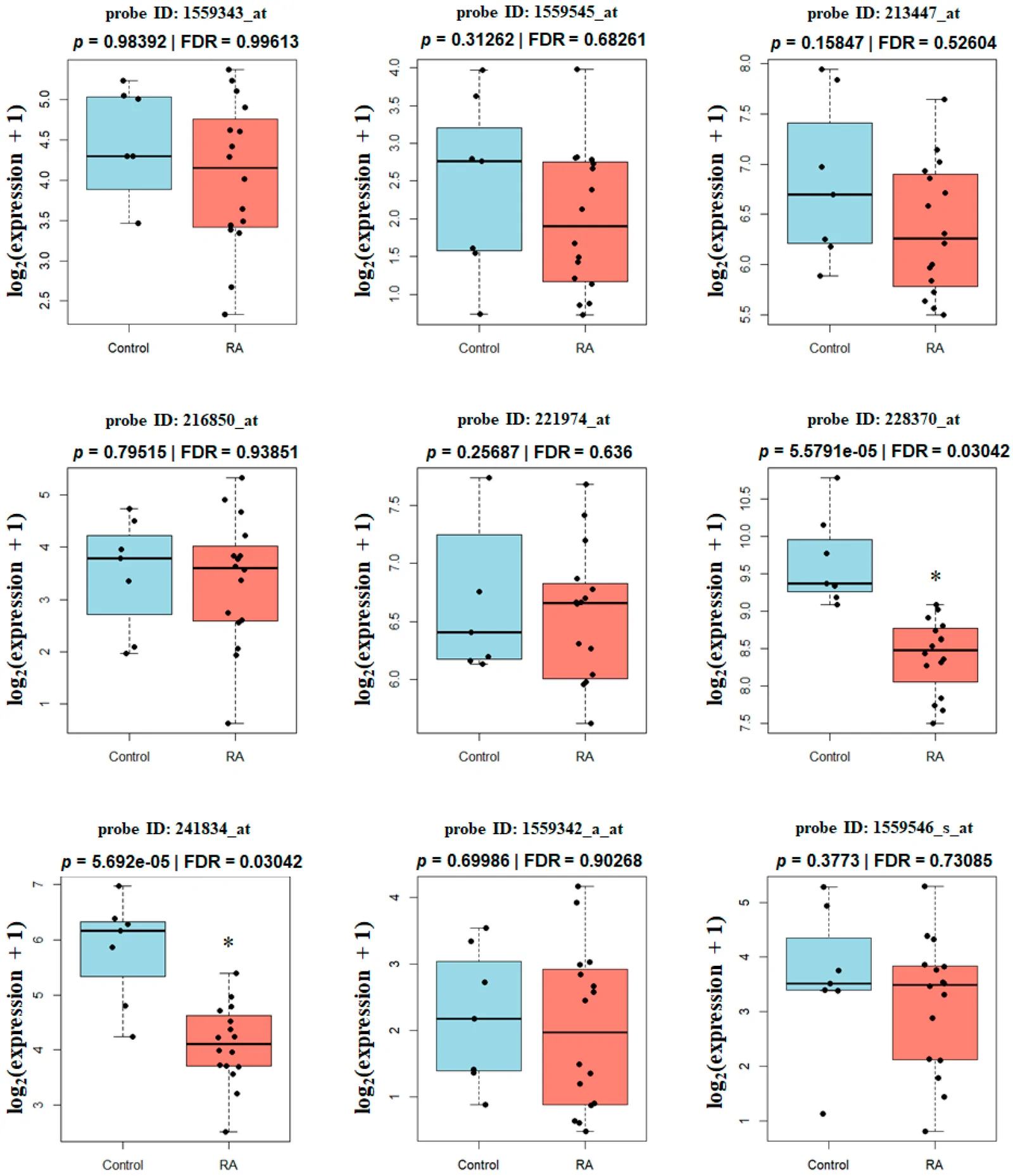
Microarray-based expression profiling of *SNHG14* across all available probe IDs in synovial tissues from RA patients (*n* = 16) and healthy controls (*n* = 7) from the GSE77298 cohort. * *p* < 0.001, empirical Bayes moderated *t*-test.

**Figure 7:**
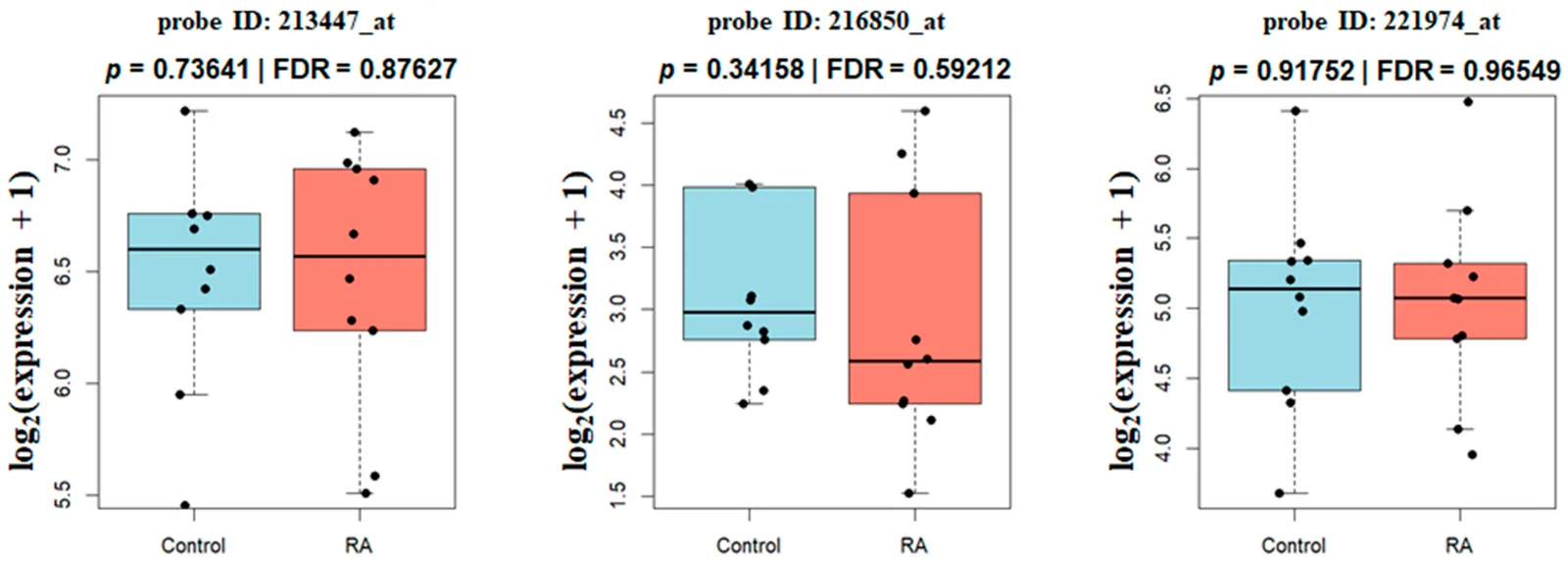
Microarray-based expression profiling of *SNHG14* across all available probe IDs in synovial tissues from RA patients (*n* = 10) and healthy controls (*n* = 10) from the GSE55235 cohort. Empirical Bayes moderated *t*-test.

**Figure 8:**
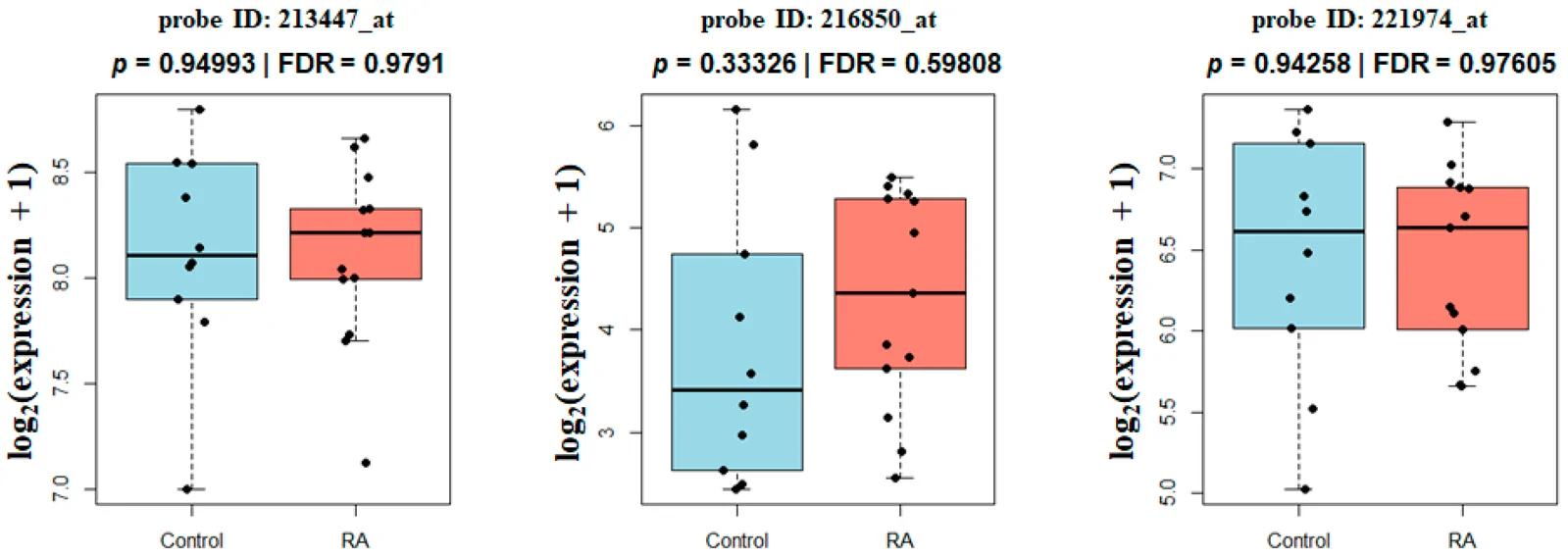
Microarray-based expression profiling of *SNHG14* across all available probe IDs in synovial tissues from RA patients (*n* = 13) and healthy controls (*n* = 10) from the GSE55457 cohort. Empirical Bayes moderated *t*-test.

For each probe ID, expression data were visualised using plots comparing only healthy controls and RA patients, where each point represents an individual patient. The upper and lower quartiles and the median are also shown (Figure 4, Figure 5, Figure 6, Figure 7 and Figure 8).

Most of the analysed probe IDs did not show statistically significant differences in gene expression between the two groups.

A statistically significant downregulation of *SNHG7* was observed in patients with RA for only one probe ID (1552729_at; *p* = 0.035). However, the result did not retain statistical significance after correction for multiple testing using the FDR (Figure 4).

Also, only one probe ID (223773_s_at) showed increased expression of *SNHG12* in RA patients (*p* = 0.039), but this was not significant after FDR correction (Figure 5).

Two probe IDs (228370_at and 241834_at) showed a statistically significant downregulation of *SNHG14* in RA patients compared to controls (*p* < 0.001), which remained significant after FDR correction (Figure 6). The remaining seven probe IDs in GSE77298 (Figure 6), three probe IDs in GSE55235 (Figure 7), and three probe IDs in GSE55457 (Figure 8) showed no statistically significant differences in expression for *SNHG14*. All results, including raw FC, expression trends, FDR, and *p*-values for the selected genes and probe IDs, are presented in Table 4.

The limitations of this study should also be noted. Each in silico dataset included a small number of samples in both the control and RA groups, which limited the statistical power. When looking at all probe IDs together, most comparisons did not reach statistical significance. There was also no clear trend toward upregulation or downregulation in either group.

## 4. Discussion

In this study, we investigated the expression of the lncRNAs *SNHG7*, *SNHG12*, and *SNHG14* in synovial membranes from patients with RA compared to a control group. We demonstrated increased expression of these lncRNAs in the synovial membranes of patients with RA. In addition, we assessed the correlations between the expression of these lncRNAs in synovial membranes and selected clinical parameters. We found a negative correlation between *SNHG14* expression and CRP levels. It appears that this correlation is inconsistent with the pro-inflammatory role of SNHG14. This correlation may be coincidental, since after applying the Bonferroni correction for multiple tests, the result is no longer statistically significant. We also cannot exclude the existence of compensatory or regulatory feedback mechanisms that lead to a decrease in CRP levels as the concentration of the pro-inflammatory SNHG14 increases.

RA is a disease with a complex pathogenesis involving numerous pro-inflammatory mediators that trigger inflammation in joint tissues. Numerous links have been demonstrated between individual mediators and the mechanisms regulating their synthesis. Studies have shown that lncRNAs play a significant role in the development of RA. LncRNAs can exert regulatory effects at the epigenetic, transcriptional, and post-transcriptional levels, as well as at the protein level [20]. It has been shown that lncRNAs can influence the functions of immune system cells involved in the development of inflammation as well as synovial cells [21,22]. Given their role in regulating gene expression—and thus in modulating the immune and inflammatory responses—lncRNAs are attracting significant interest in the context of monitoring the clinical course of RA. A better understanding of the role of lncRNAs in the pathogenesis of RA may prove helpful in the search for new diagnostic biomarkers. An integrative strategy combining bioinformatics predictions with experimental studies may provide valuable guidance for the development of clinical and research strategies for the treatment of RA [23].

Previous studies suggest that lncRNAs *SNHG7*, *SNHG12*, and *SNHG14* are involved in regulating several processes that may contribute to the development of inflammation and affect cartilage and bone metabolism. Most studies on the role of lncRNAs have been performed on cellular or animal models. To date, very few clinical studies have been carried out to evaluate the role of the *SNHG7*, *SNHG12*, and *SNHG14* genes in the pathogenesis of RA. There are also some studies on the role of lncRNAs in the pathogenesis of osteoarthritis (OA), which, however, differs pathophysiologically from rheumatoid arthritis [24,25,26]. Lin et al. [27] investigated the effect of *SNHG7* on chondrocyte function in a mouse model of OA. The authors demonstrated that *SNHG7* exerts a protective and anti-inflammatory effect on cartilage tissue. *SNHG7* improved cartilage structure, increased chondrocyte proliferation, and inhibited apoptosis as well as the release of interleukin-1 beta (IL-1β) in chondrocytes derived from mice with OA that had been treated with IL-1β. Another study [28] showed that *SNHG7* plays an important role in proliferation, apoptosis, and autophagy in OA. *SNHG7* inhibited chondrocyte apoptosis and autophagy and increased chondrocyte proliferation in inflammatory damage, oxidative stress, and ferroptosis in IL-1β-induced chondrocytes. The authors of another study [29] have shown decreased expression of *SNHG7* in the cartilage of OA patients. Moreover, it was shown that *SNHG7* exerted an anti-inflammatory effect by inhibiting the p38 MAPK signalling pathway, resulting in decreased concentrations of tumour necrosis factor-alpha (TNF-α) and IL-6, reduced apoptosis, and increased cell viability. In the Chinese population, reduced expression of the *SNHG7* gene has been demonstrated in patients with osteoarthritis [30]. Furthermore, *SNHG7* increased cell viability and inhibited apoptosis and inflammation in chondrocytes exposed to IL-1β.

To date, most studies on the role of *SNHG12* have focused on cancer. Very few studies have examined the effects of this lncRNA on cartilage and bone tissue function. It has been demonstrated that *SNHG12* expression was elevated in tissues affected by OA [31]. Furthermore, IL-1β induced the expression of the lncRNA *SNHG12* in chondrocytes. *SNHG12* also inhibited chondrocyte proliferation by exacerbating inflammation and apoptosis and by increasing extracellular matrix degradation.

The studies investigating the role of *SNHG14* in pro-inflammatory cytokine production in RA indicated that downregulation of *SNHG14* inhibited the proliferation of activated THP-1 cells and the production of pro-inflammatory cytokines. *SNHG14* enhanced Jun N-terminal kinase (JNK) signalling via the miR-17-5p/MINK1 axis, thereby activating the JNK pathway through MINK1 and amplifying the inflammatory response [32]. The authors of another study [33] demonstrated the pro-inflammatory effects of *SNHG14* and increased *SNHG14* expression in tissues affected by OA. Downregulation of *SNHG14* enhanced chondrocyte proliferation and inhibited cell apoptosis as well as the expression of cyclooxygenase-2 (*COX-2*), inducible nitric oxide synthase (*iNOS*), *TNF-α*, and *IL-6*. Furthermore, it was shown that reducing *SNHG14* expression alleviated the progression of OA in rats by inhibiting inflammatory responses. *SNHG14* has also been shown to have a pro-inflammatory effect in cartilage tissue. Blocking *SNHG14* increased chondrocyte viability, inhibited their apoptosis, and inhibited extracellular matrix degradation [34]. It has been demonstrated that serum *SNHG14* levels increase in patients with acute gouty arthritis, and blocking *SNHG14* inhibits the inflammatory response [35].

To compare our results with those of other researchers, we performed an in silico analysis, including three public datasets (GSE77298 [18], GSE55235 [19], and GSE55457 [19]) sourced from the Gene Expression Omnibus (GEO) database using the GEOquery R package. In the case of SNHG12, the results found in the available databases were consistent with ours. In both cases, increased expression of SNHG12 was observed in patients with RA. For SNHG7 and SNHG14, the available databases showed a trend toward lower expression of these lncRNAs in patients with RA; however, these differences did not reach statistical significance. The observed differences may be due to the small number of patients included in the available databases. Other possible causes of the observed differences may include disease duration and activity, treatment status, or even differences in the cellular composition of the tissue being studied.

Finally, it is important to note a few limitations of this study. First, the study was limited to assessing changes in SNHG expression at the mRNA level, supplemented by in silico analyses. Experimental validation of target proteins and signalling pathways was not included. An additional limitation concerns the in silico analyses, which were based on publicly available datasets comprising relatively small groups of patients. Furthermore, the patients included in these datasets may differ from the population in our study in terms of disease activity, treatment history, and other clinical parameters. Therefore, the results of the in silico analyses should be interpreted with caution.

In addition, although the use of a single reference gene to normalize qPCR results constitutes a methodological limitation, we believe that RPLP0 is the most appropriate choice for this study, given the highly heterogeneous nature of synovial tissue in RA and its relatively stable expression compared to alternative reference genes, such as beta-2-microglobulin. Despite all these limitations, we hope that the results of our study will spark greater interest among researchers in the role of lncRNAs in the pathogenesis of RA.

## 5. Conclusions

To date, studies have indicated that the lncRNAs *SNHG7*, *SNHG12*, and *SNHG14* are involved in regulating several processes that affect cartilage and bone metabolism, as well as immune system function. At this stage, studies are too few to determine the exact role of these lncRNAs in RA. The elevated levels of these lncRNAs detected in our study among patients with RA may suggest their possible involvement in disease development. The lack of statistically significant correlations between the lncRNAs studied and clinical parameters of RA seems to suggest that they are not significant regulators of disease activity. However, understanding their exact role in the pathogenesis of RA requires further investigation.

## Figures and Tables

**Table 1:** Correlations between *SNHG14* expression in synovial membranes and clinical parameters in the RA group.

Parameters	Rs	*p*
Age (years)	−0.0396	0.7941
Disease duration (years)	0.1421	0.3463
BMI (kg/m^2^)	−0.1658	0.2707
VAS	0.0115	0.9396
DAS28	−0.2546	0.0877
Larsen score	0.2060	0.1696
Tender joint count	−0.2429	0.1039
Swollen joint count	−0.2541	0.0883
CRP (mg/L)	−0.3971	* 0.0063
ESR (mm/h)	−0.1349	0.3715
Average global grip strength (kg)	−0.0858	0.5708
Average two-point grip strength (kg)	−0.0365	0.8097

R_s_—Spearman rank correlation coefficient; * *p* < 0.05. Abbreviations: BMI—body mass index; VAS—Visual Analogue Scale; DAS28—Disease Activity Score in 28 joints; CRP—C-reactive protein; ESR—erythrocyte sedimentation rate.

**Table 2:** Correlations between *SNHG7* expression in synovial membranes and clinical parameters in the RA group.

Parameters	Rs	*p*
Age (years)	−0.0499	0.7421
Disease duration (years)	0.0600	0.6918
BMI (kg/m^2^)	−0.0830	0.5834
VAS	0.0953	0.5289
DAS28	−0.0986	0.5143
Larsen score	0.0021	0.9889
Tender joint count	−0.0926	0.5403
Swollen joint count	−0.1353	0.3698
CRP (mg/L)	−0.2411	0.1064
ESR (mm/h)	−0.0923	0.5420
Average global grip strength (kg)	−0.1045	0.4895
Average two-point grip strength (kg)	−0.0463	0.7601

R_s_—Spearman rank correlation coefficient. Abbreviations: BMI—body mass index; VAS—Visual Analogue Scale; DAS28—Disease Activity Score in 28 joints; CRP—C-reactive protein; ESR—erythrocyte sedimentation rate.

**Table 3:** Correlations between *SNHG12* expression in synovial membranes and clinical parameters in the RA group.

Parameters	Rs	*p*
Age (years)	0.0793	0.6003
Disease duration (years)	−0.1466	0.3310
BMI (kg/m^2^)	−0.1220	0.4193
VAS	−0.1358	0.3681
DAS28	−0.2335	0.1184
Larsen score	−0.0398	0.7928
Tender joint count	−0.1957	0.1924
Swollen joint count	0.0522	0.7303
CRP (mg/L)	−0.1331	0.3779
ESR (mm/h)	−0.1669	0.2676
Average global grip strength (kg)	0.0086	0.9545
Average two-point grip strength (kg)	0.1715	0.2544

R_s_—Spearman rank correlation coefficient. Abbreviations: BMI—body mass index; VAS—Visual Analogue Scale; DAS28—Disease Activity Score in 28 joints; CRP—C-reactive protein; ESR—erythrocyte sedimentation rate.

**Table 4:** In silico differential expression analysis of *SNHG7*, *SNHG12*, and *SNHG14* genes based on three independent GEO datasets [18,19].

GEO Accession	Gene	Probe ID	LFC	Raw Fold Change	Fold Difference	*p*-Value	FDR-Value	Expression Trend
GSE77298	*SNHG7*	229050_s_at	−0.297	0.814	−1.23	0.423	0.760	D
		1552729_at	−0.959	0.514	−1.94	* 0.035	0.284	D
	*SNHG12*	223773_s_at	0.639	1.558	1.56	* 0.039	0.300	U
		223774_at	0.748	1.680	1.68	0.107	0.451	U
		228990_at	0.396	1.316	1.32	0.139	0.499	U
	*SNHG14*	1559343_at	0.010	1.007	1.01	0.984	0.996	U
		1559545_at	−0.454	0.730	−1.37	0.313	0.683	D
		213447_at	−0.467	0.723	−1.38	0.158	0.526	D
		216850_at	−0.133	0.912	−1.1	0.795	0.939	D
		221974_at	−0.444	0.735	−1.36	0.257	0.636	D
		228370_at	−1.264	0.416	−2.4	* 0.000056	** 0.030	D
		241834_at	−1.714	0.305	−3.28	* 0.000057	** 0.030	D
		1559342_a_at	−0.195	0.874	−1.14	0.700	0.903	D
		1559546_s_at	−0.477	0.719	−1.39	0.377	0.731	D
GSE55235	*SNHG14*	213447_at	−0.087	0.942	−1.06	0.736	0.876	D
		216850_at	0.427	0.744	−1.34	0.342	0.592	D
		221974_at	0.033	1.023	1.02	0.918	0.965	U
GSE55457	*SNHG14*	213447_at	−0.012	0.991	−1.01	0.950	0.979	D
		216850_at	0.473	1.388	1.39	0.333	0.598	U
		221974_at	−0.020	0.986	−1.01	0.943	0.976	D

* *p* < 0.05; ** FDR < 0.05. Abbreviations: LFC—Log_2_ fold change; 2^LFC^—raw fold change; FDR—false discovery rate; U—upregulation; D—downregulation.

## Data Availability

Data are available upon reasonable request.

## Supplementary Materials

The following supporting information can be downloaded at https://www.mdpi.com/article/10.3390/cimb48090860/s1.
