# Supplementary material for: Long Non-Coding RNAs SNHG7, SNHG12, and SNHG14 in Rheumatoid Arthritis: Clinical and In Silico Analysis
Source: Curr Issues Mol Biol. 2026 Aug 25;48(9):860. doi: 10.3390/cimb48090860 (PMC13605108; doi:10.3390/cimb48090860)
Supplement: Supplementary file 1 [file cimb-48-00860-s001.zip › cimb-4472381-supplementary.pdf]

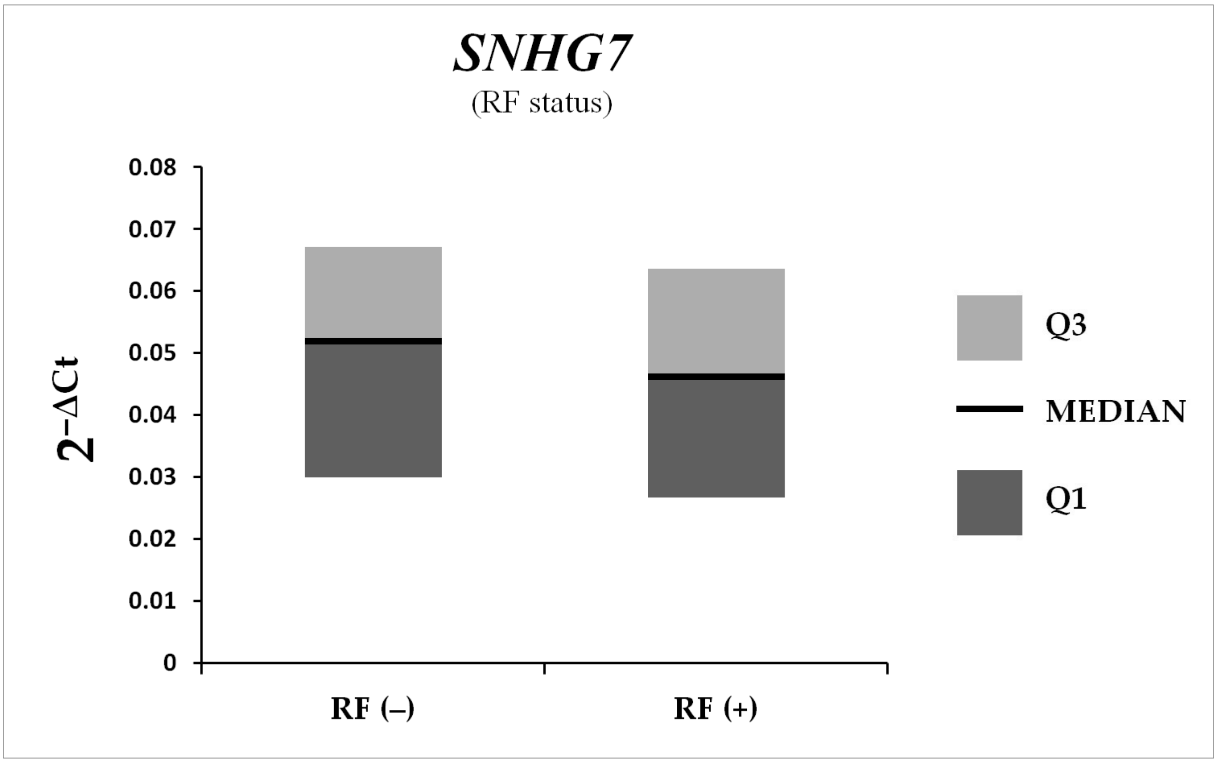

Figure S1. *SNHG7* expression in synovial tissue of RA patients according to rheumatoid factor serostatus (RF+ and RF–)

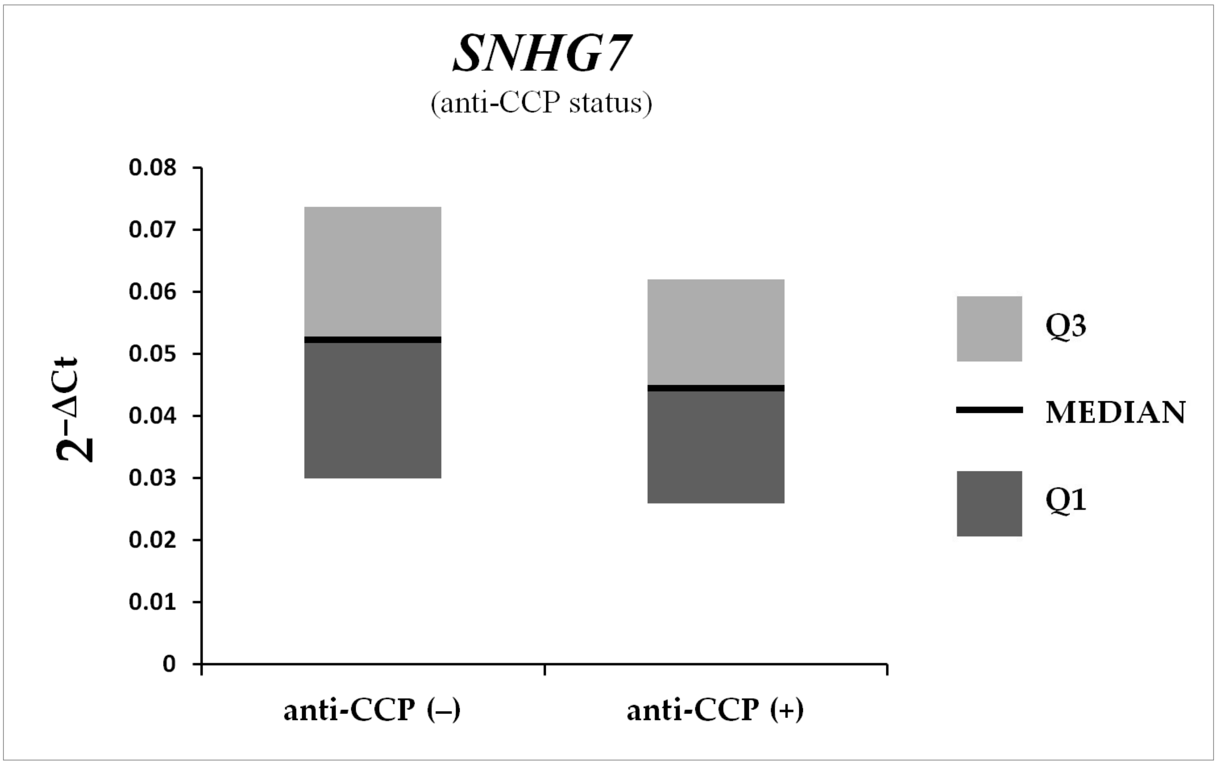

Figure S2. *SNHG7* expression in synovial tissue of RA patients according to anti-CCP antibody status (anti-CCP+ and anti-CCP−)

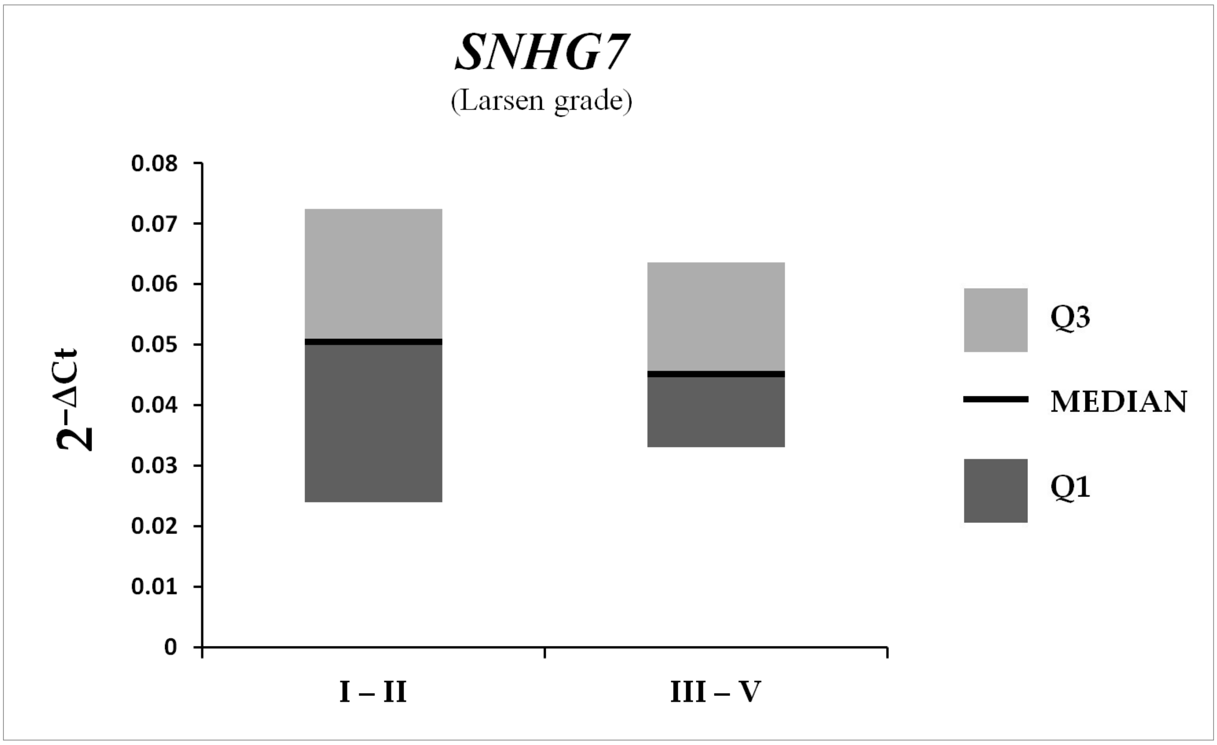

Figure S3. *SNHG7* expression in synovial tissue of RA patients according to radiographic severity (Larsen stages I–II vs. III–V)

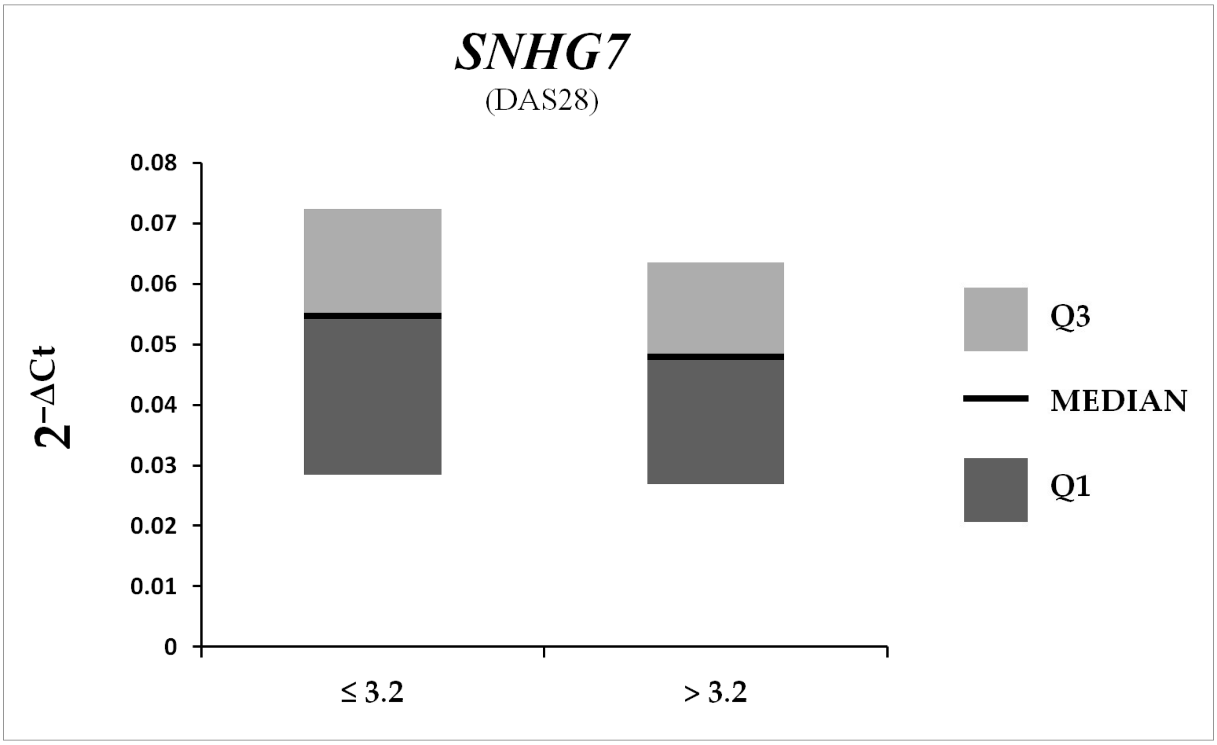

Figure S4. *SNHG7* expression in synovial tissue of RA patients according to disease activity DAS28 (≤3.2 and >3.2)

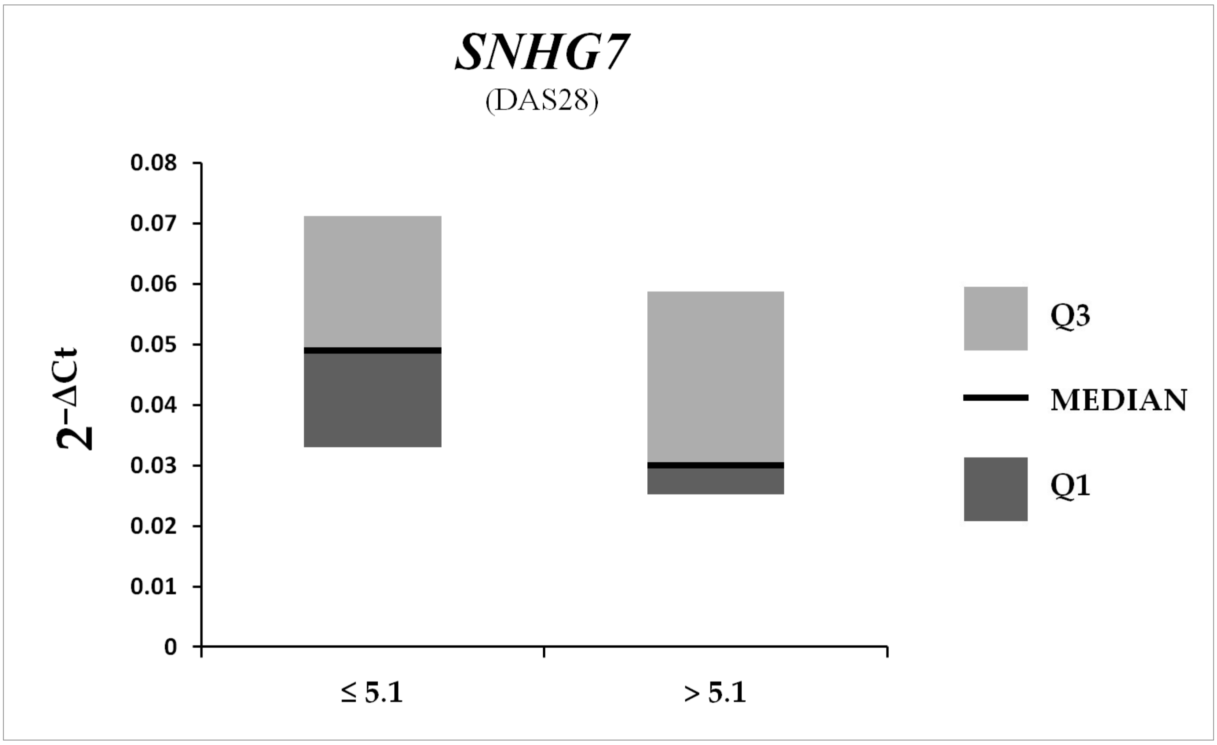

Figure S5. *SNHG7* expression in synovial tissue of RA patients according to disease activity DAS28 (≤5.1 and >5.1)

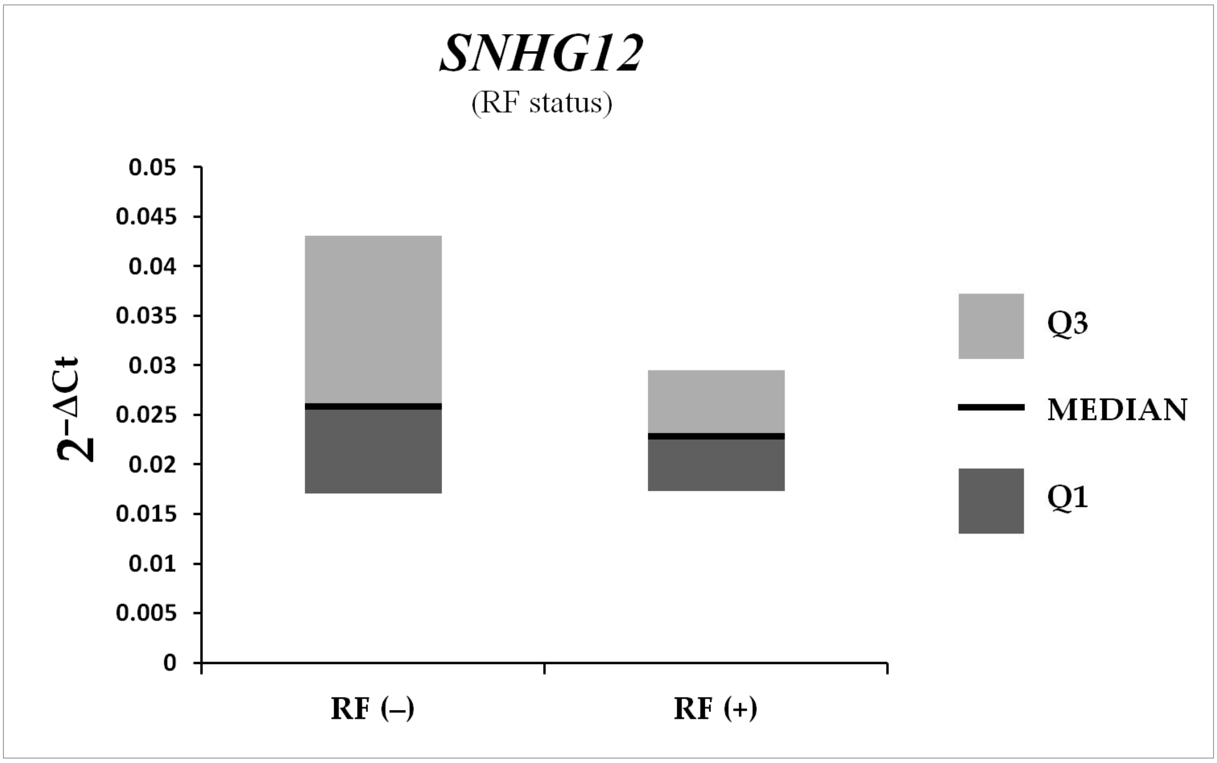

Figure S6. *SNHG12* expression in synovial tissue of RA patients according to rheumatoid factor serostatus (RF+ and RF–)

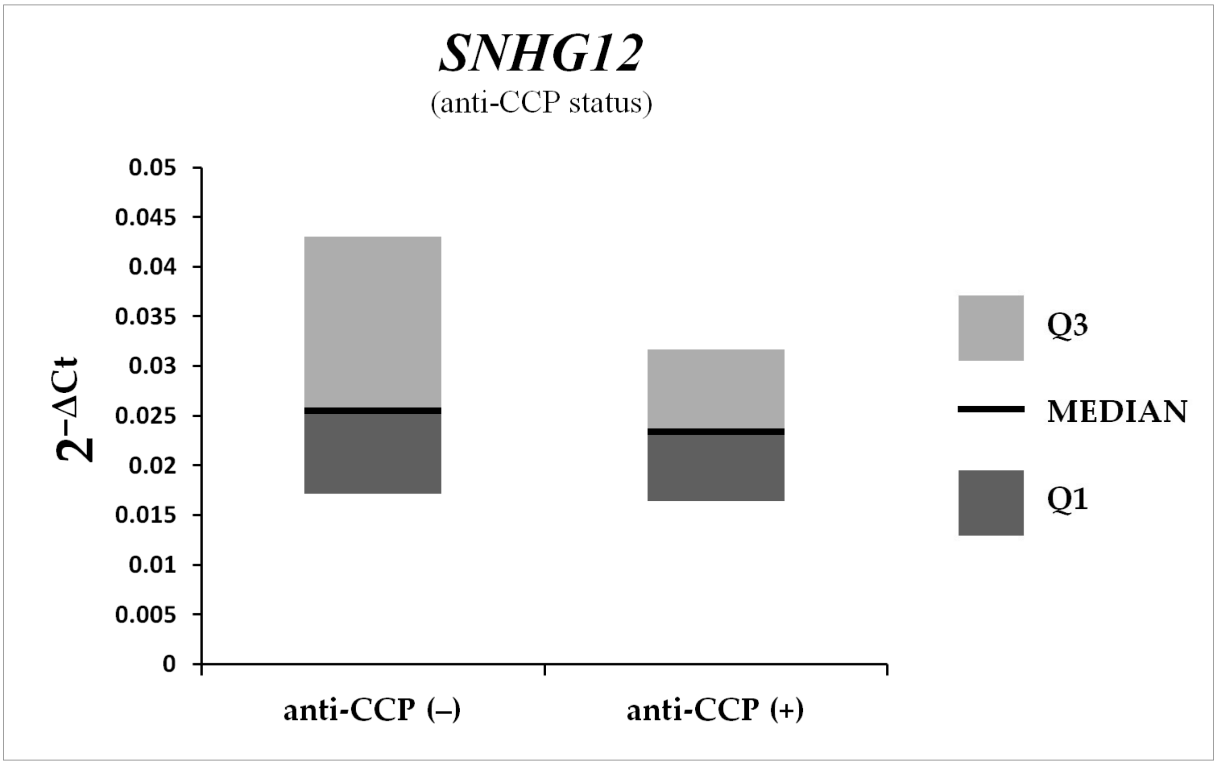

Figure S7. *SNHG12* expression in synovial tissue of RA patients according to anti-CCP antibody status (anti-CCP+ and anti-CCP−)

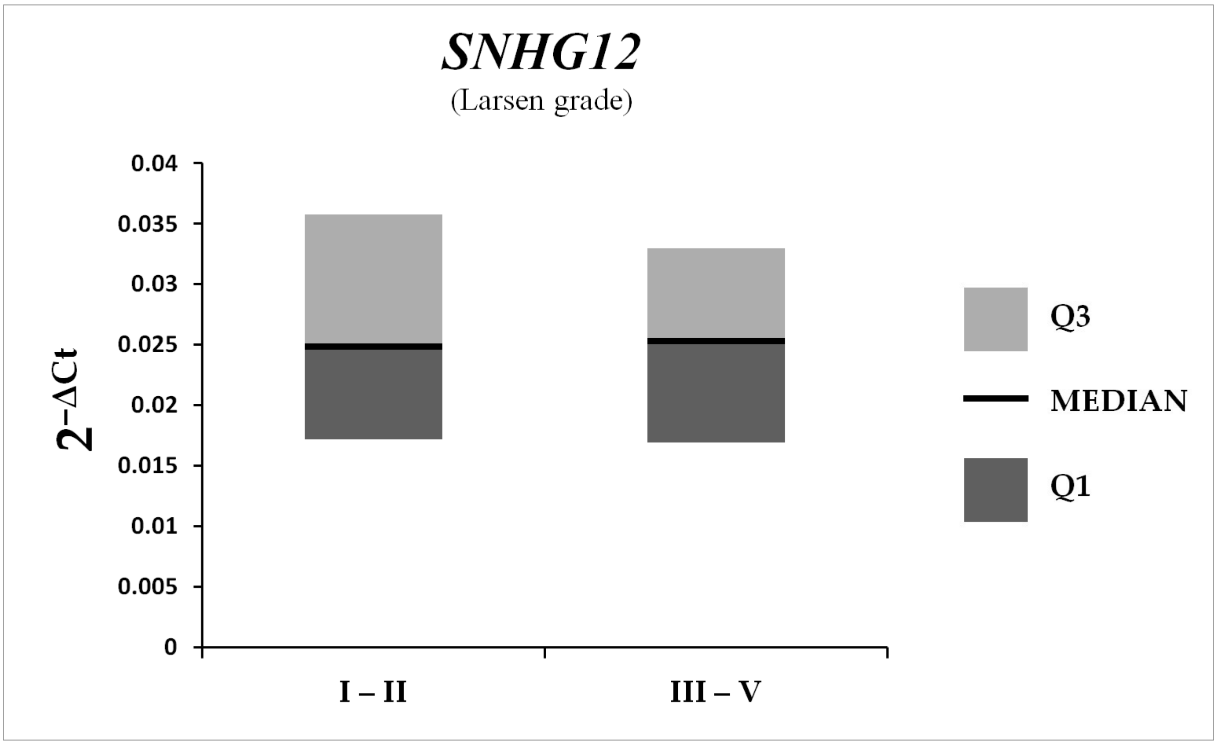

Figure S8. *SNHG12* expression in synovial tissue of RA patients according to radiographic severity (Larsen stages I–II vs. III–V)

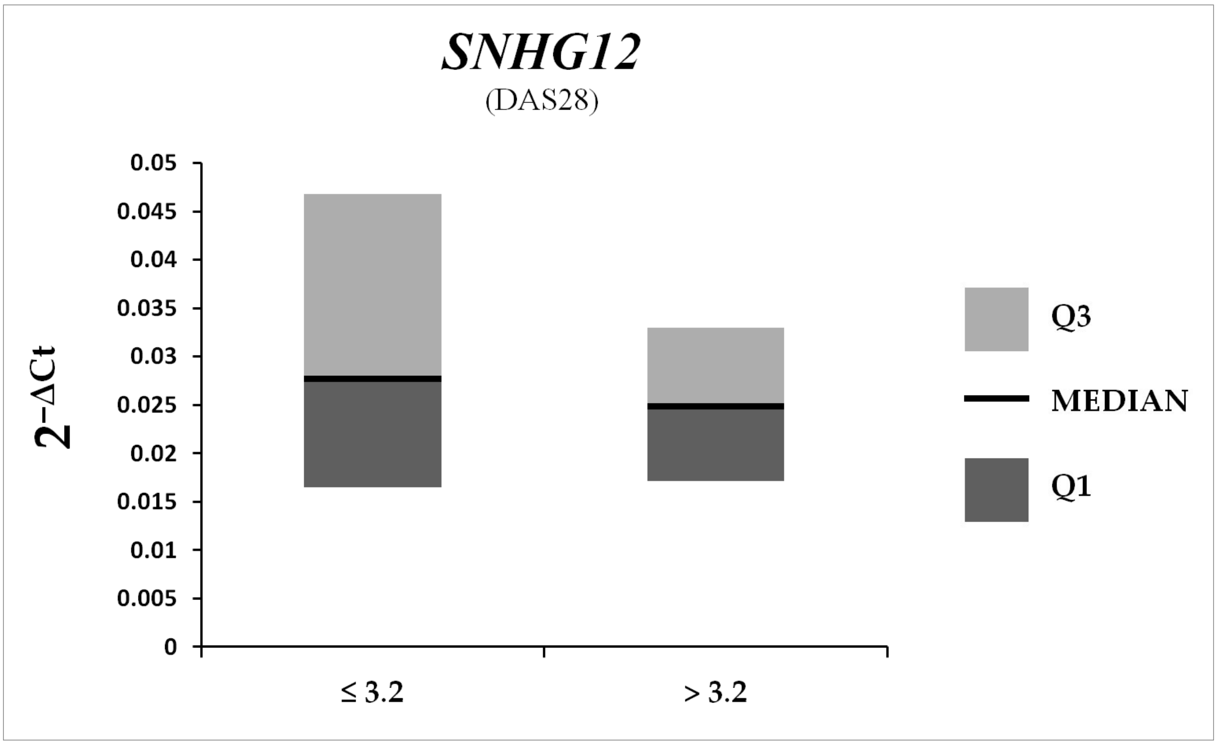

Figure S9. *SNHG12* expression in synovial tissue of RA patients according to disease activity DAS28 (≤3.2 and >3.2)

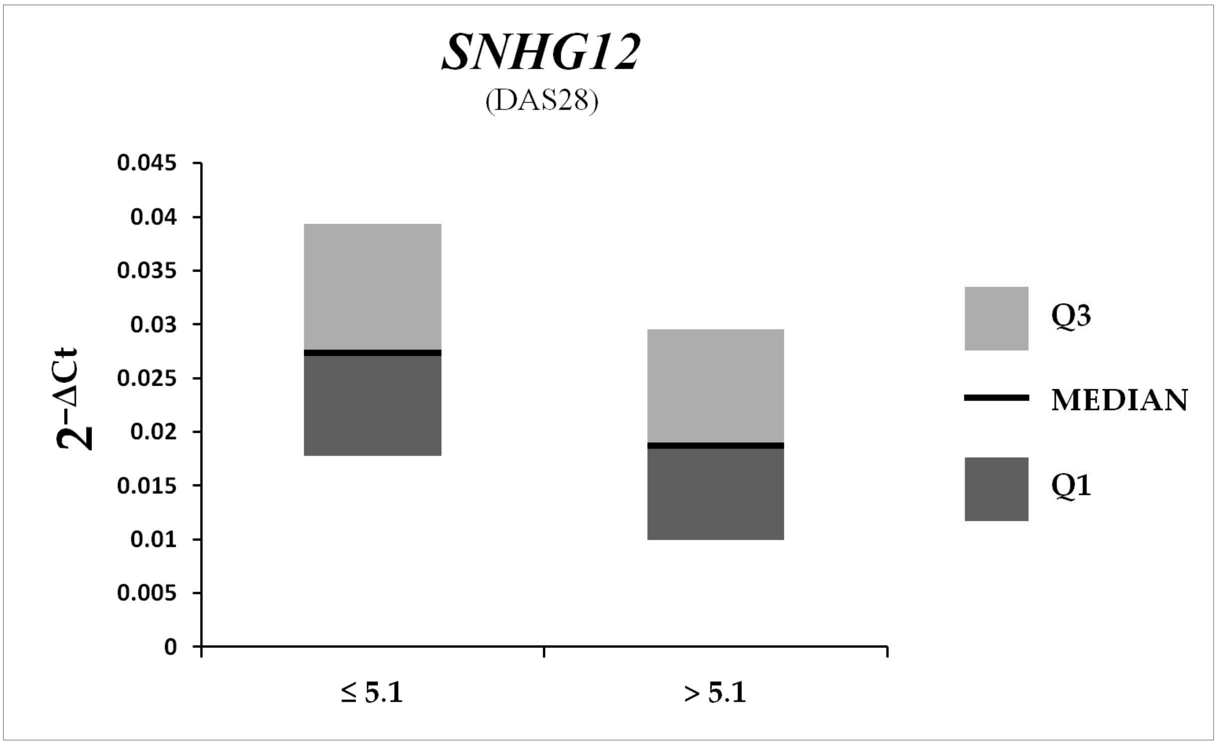

Figure S10. *SNHG12* expression in synovial tissue of RA patients according to disease activity DAS28 (≤5.1 and >5.1)

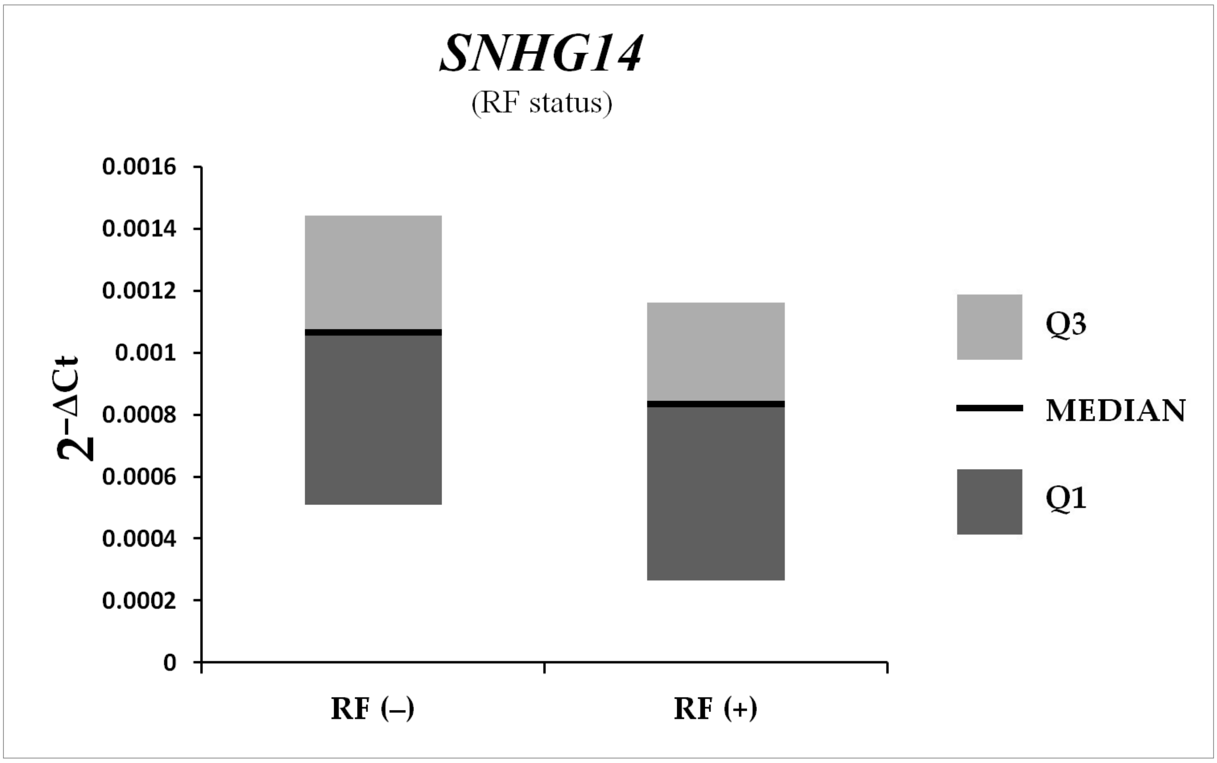

Figure S11. *SNHG14* expression in synovial tissue of RA patients according to rheumatoid factor serostatus (RF+ and RF–)

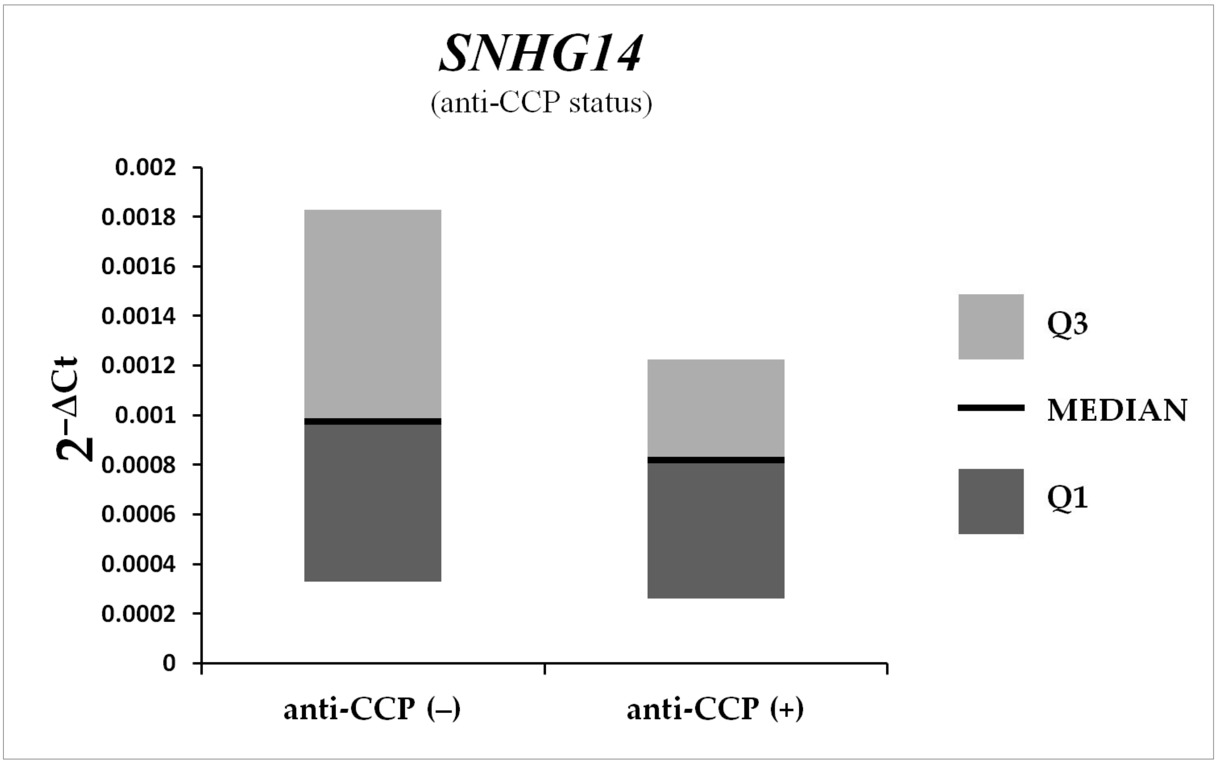

Figure S12. *SNHG14* expression in synovial tissue of RA patients according to anti-CCP antibody status (anti-CCP+ and anti-CCP−)

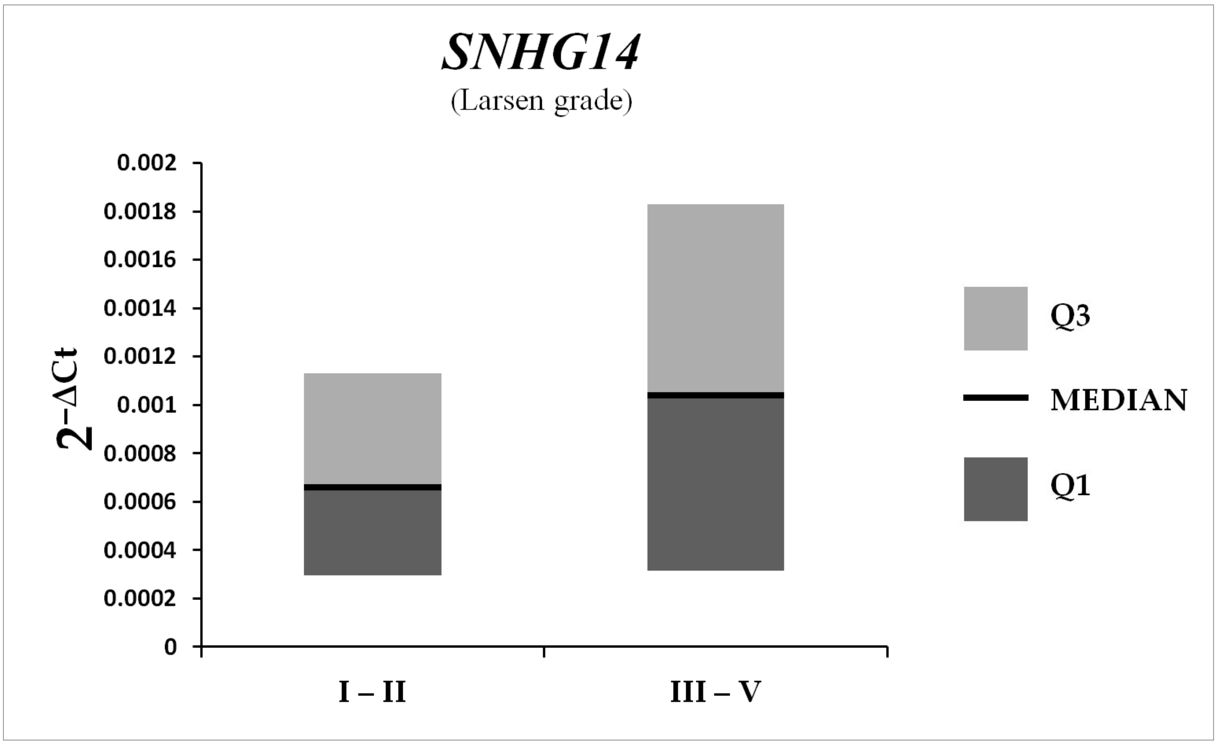

Figure S13. *SNHG14* expression in synovial tissue of RA patients according to radiographic severity (Larsen stages I–II vs. III–V)

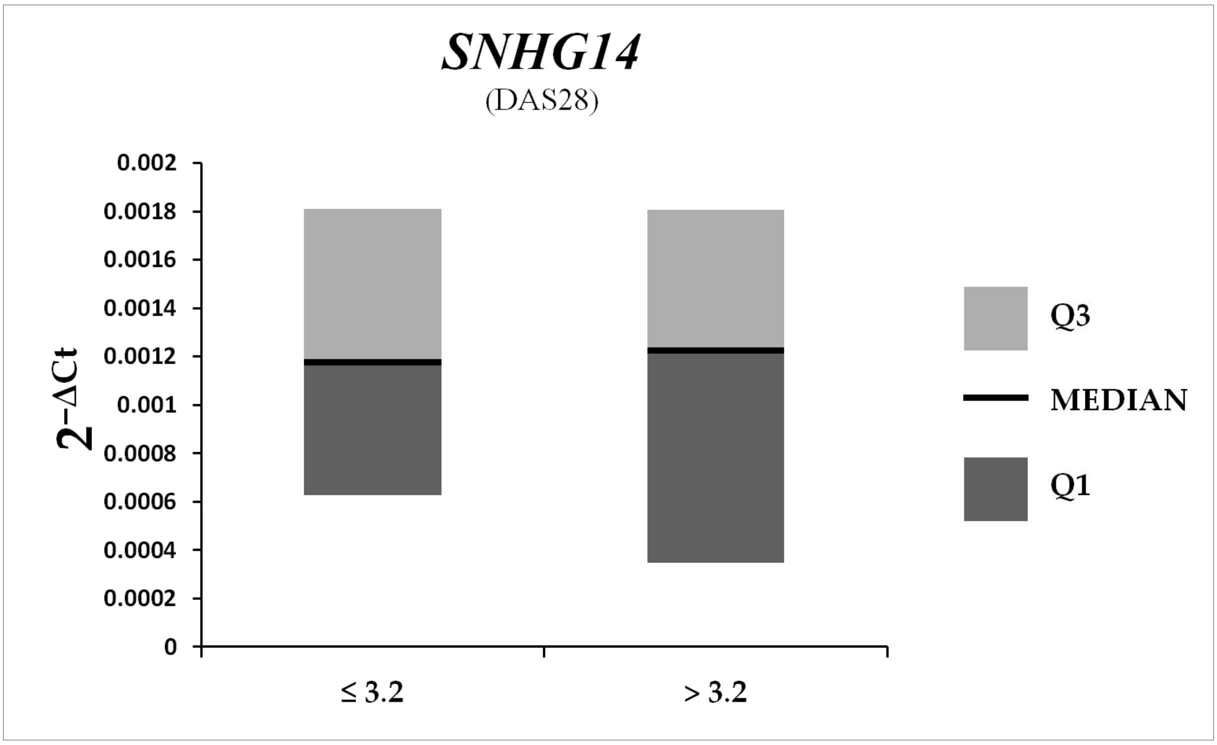

Figure S14. *SNHG14* expression in synovial tissue of RA patients according to disease activity DAS28 (≤3.2 and >3.2)

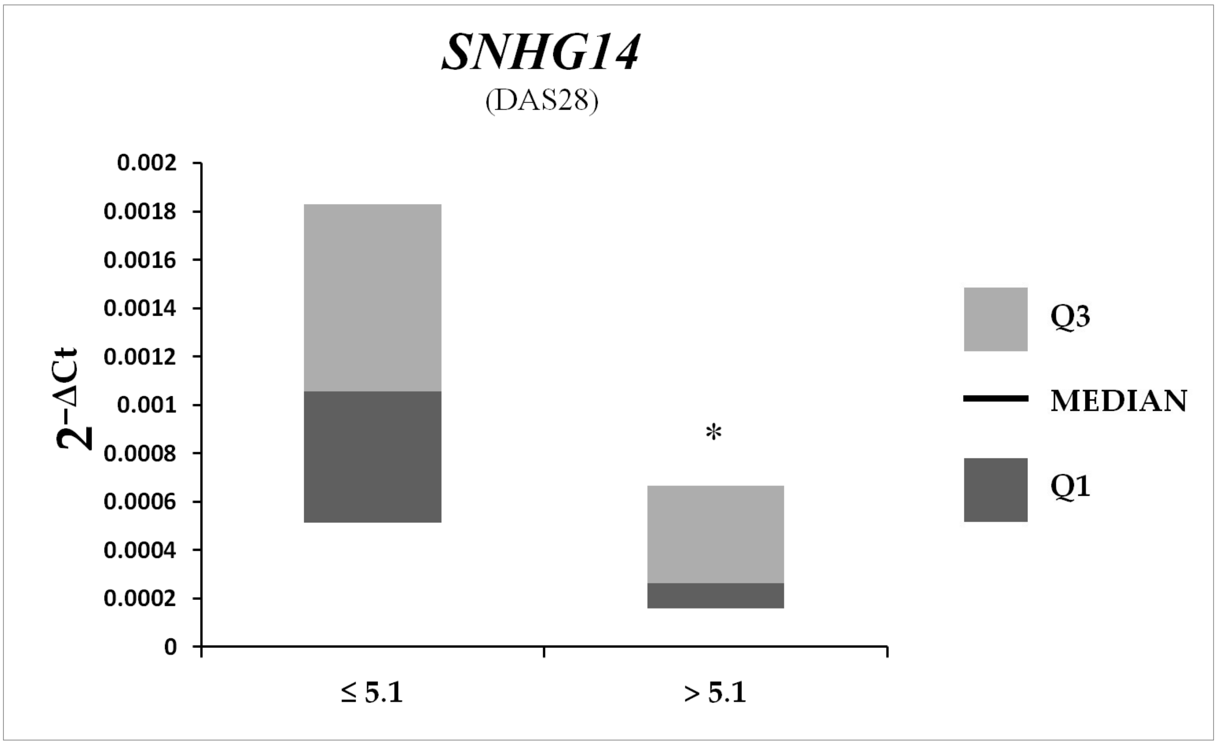

Figure S15. *SNHG14* expression in synovial tissue of RA patients according to disease activity DAS28 (≤5.1 and >5.1), * $p = 0.004$, Mann–Whitney U test
